# 63565 Awareness of Low Value Care Is Requisite for De-Implementation: Nurses’ Choosing Wisely ®Campaign

**DOI:** 10.1017/cts.2021.539

**Published:** 2021-03-30

**Authors:** Kathleen R. Stevens

**Affiliations:** University of Texas Health San Antonio for the Texas Team on The Future of Nursing

## Abstract

ABSTRACT IMPACT: Points to strategies to de-implement ineffective, harmful, or unproven practices, lowering burden and cost of healthcare, using evidence-based recommendations on low value care. OBJECTIVES/GOALS: Ineffective, harmful, or unproven practices add burden and cost of healthcare. In national efforts to de-implement low value care (LVC), Choosing Wisely ®campaign generated 25 recommendations through the American Academy of Nursing (CW AAN). Our study described nurse-awareness of CW AAN recommendations as requisite toward de-implementing LVC. METHODS/STUDY POPULATION: A multi-stakeholder state action coalition led the project to achieve the Institute of Medicine Future of Nursing goals by describing nurse awareness of CW AAN recommendations. The survey was the first among nursing professionals. Use of human subjects was approved at the lead university. Registered Nurse contact information was obtained from the state Board of Nursing of a large mid-South state. Qualtrics ®surveys patterned after the CW survey of physicians’ awareness were administered online by the state Center for Nursing Workforce Studies. Content experts developed 2 surveys’‘ one for Registered Nurses (RNs) and one for Advance Practice Registered Nurses (APRNs)’‘ to account for differences in scope of practice. Surveys assessed current knowledge and perception of the Choosing Wisely ®AAN campaign. RESULTS/ANTICIPATED RESULTS: Over six weeks, 374 nurses participated (295 RNs and 79 APRNs). About half of each group indicated that unnecessary nursing care was a ‘somewhat serious problem.’ Only 21% of RNs and 26% of APRNs were aware of Choosing Wisely ®AAN recommendations. Participants identified reasons for the prevalence of low value care in practice as being concerns about malpractice issues, lack of time with patients for meaningful discussion, ‘just to be safe,’ and patients insisting on getting the test or procedure. For the RN group, cost of LVC was rarely discussed; in the APRN group, cost was frequently discussed. Of the APRNs who were aware of CW, 90% believe the recommendations were helpful. When asked for LVC de-implementation suggestions, 78% said EBP recommendations would be effective; at the same time, 20% had low knowledge of EBP. DISCUSSION/SIGNIFICANCE OF FINDINGS: RNs and APRNs reported low awareness of CW AAN advice. While representative, sample size limits generalization. De-implementation in learning health systems will include socioecological strategies focused on provider awareness and confidence, patient preference, cost, strength of evidence, and safe work culture to diffuse fear of litigation.

